# Supplementary material for: Abrupt hippocampal remapping signals resolution of memory interference
Source: Nat Commun. 2021 Aug 10;12:4816. doi: 10.1038/s41467-021-25126-0 (PMC8355182; doi:10.1038/s41467-021-25126-0)
Supplement: Supplementary file 1 — Supplementary information [file 41467_2021_25126_MOESM1_ESM.pdf]

Supplementary information

Supplementary Table 1. Number of scene pairmates that transitioned to 'learned' status at each round and for each participant. 'Learned' was defined as high-confidence, correct associative memory for both pairmates. The round at which pairmates transitioned to 'learned' is referred to as the 'learned round' (LR). Note: pairmates that were learned in the first round or never learned were excluded from fMRI analyses.

Participant # \ Round	1	2	3	4	5	6	Never Learned
**1**	1	7	6	4	0	0	0
**2**	1	1	4	4	6	2	0
**3**	1	7	5	5	0	0	0
**4**	0	3	0	5	4	3	3
**5**	0	2	3	6	4	2	1
**6**	3	6	2	6	0	1	0
**7**	0	6	4	3	3	1	1
**8**	0	2	5	4	5	1	1
**9**	0	1	1	2	2	2	10
**10**	0	0	8	2	5	2	1
**11**	3	3	4	3	2	2	1
**12**	0	1	2	5	2	5	3
**13**	1	1	2	4	7	2	1
**14**	0	0	3	4	4	5	2
**15**	1	6	7	2	1	1	0
**16**	1	2	6	1	2	4	2
**17**	2	3	3	5	3	2	0
**18**	5	3	2	3	4	0	1
**19**	0	0	2	7	6	2	1
**20**	0	1	6	2	1	4	4
**21**	0	1	3	3	4	7	0
**22**	1	3	4	2	3	1	4
**23**	0	6	5	4	1	2	0
**24**	3	4	7	1	2	1	0
**25**	1	10	4	3	0	0	0
**26**	0	0	2	9	2	1	4
**27**	3	0	4	2	2	1	6
**28**	1	8	4	3	0	0	2
**29**	0	6	2	1	1	2	6
**30**	2	6	6	1	0	2	1
**31**	1	1	3	6	3	3	1

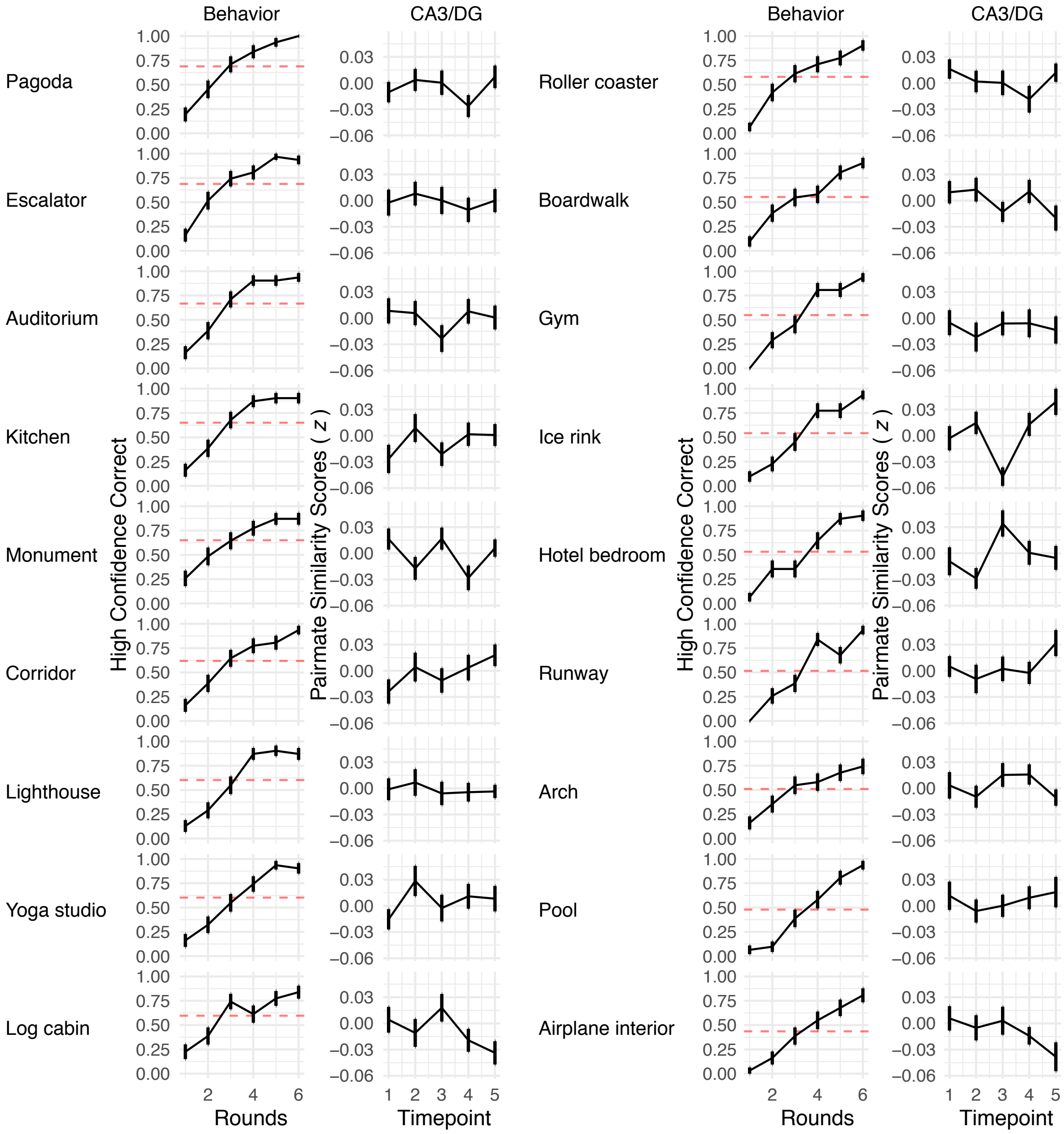

Supplementary Figure 1. Behavioral accuracy and CA3/dentate gyrus pairmate similarity scores for each set of scene pairmates. Scene pairmates are rank-ordered from highest (top, left column) to lowest (bottom, right column) mean accuracy on the associative memory test rounds. Individual plots of 'behavior' show mean associative memory test accuracy by learning round (1-6) for each set of scene pairmates. Mean test accuracy across all rounds is denoted by the red dashed line. A repeated measures ANOVA with factors of stimulus pair and round revealed a significant main effect of stimulus pair ($F_{17,510} = 4.08$, $p < 0.001$, $\eta^2 = 0.08$) as well as a significant interaction between stimulus pair and round ($F_{17,510} = 2.18$, $p = 0.004$, $\eta^2 = 0.02$). Individual plots of 'CA3/DG' show mean pairmate similarity scores in CA3/dentate gyrus by timepoint (1-5) for each set of scene pairmates. Each timepoint reflects similarity in CA3/dentate gyrus across successive learning rounds (1-2, 2-3, etc.). A repeated measures ANOVA with factors of timepoint and stimulus pair did not reveal a significant main effect of stimulus pair ($F_{17,510} = 0.74$, $p = 0.760$, $\eta^2 = 0.01$) or an interaction between stimulus pair and timepoint in ($F_{17,510} = 1.49$, $p = 0.093$, $\eta^2 = 0.02$). Note: data are presented as mean values ± S.E.M., $n = 31$ independent participants. Source data are provided as a Source Data file.

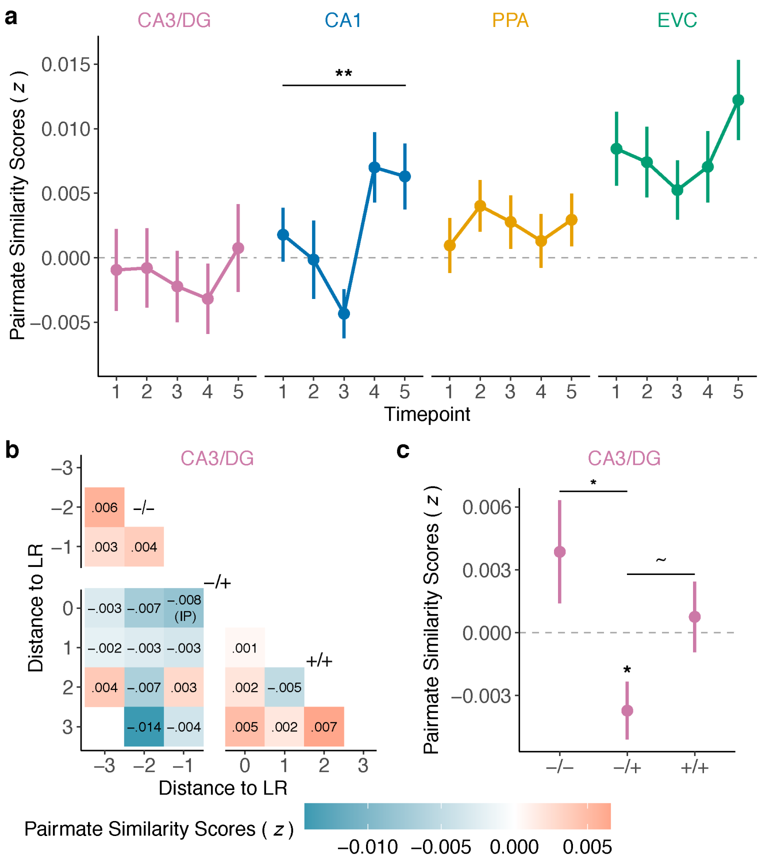

Supplementary Figure 2. Pairmate similarity scores as a function of timepoints and learning. a. Pairmate similarity scores at each timepoint for each region of interest (ROI). Each timepoint reflects correlations between successive scene exposure rounds [i.e., timepoint 1 = *r*(round 1, round 2), timepoint 2 = *r*(round 2, round 3), etc.]. CA1 is the only ROI that showed a significant main effect of timepoint (CA3/DG: $F_{4,120} = 0.24$, $p = 0.913$, $\eta^2 = 0.006$; CA1: $F_{4,120} = 3.89$, $p = 0.005$, $\eta^2 = 0.09$; PPA: $F_{4,120} = 0.34$, $p = 0.848$, $\eta^2 = 0.01$; EVC: $F_{4,120} = 0.82$, $p = 0.517$, $\eta^2 = 0.02$; repeated measures ANOVAs). **b.** Pairmate similarity scores in CA3/dentate gyrus (CA3/DG) calculated by correlating all possible combinations of the scene exposure rounds, expressed in terms of distance relative to the learned round (LR) for each pairmate. Rounds that preceded the LR reflect rounds before learning occurred, whereas the LR and following rounds reflect rounds after learning occurred (i.e., high confidence correct performance on the associative memory test). Thus, the correlations can be grouped into 3 categories: correlations among 'before' rounds (-/-), correlations between 'before' and 'after' rounds (-/+), and correlations among 'after' rounds (+/+). **c.** CA3/dentate gyrus pairmate similarity scores averaged across all of the cells within each of the three categories (-/-, -/+, +/+). Pairmate similarity scores in the -/+ category were significantly below 0 ($t_{30} = -2.70$, $p = 0.011$, $d = 0.48$, CI = [-0.004 ± 0.003], two-tailed one sample *t*-test) and significantly lower than the -/- category ($t_{30} = 2.49$, $p = 0.018$, $d = 0.45$, CI = [0.008 ± 0.006], two-tailed paired samples *t*-test). There was a trend toward lower pairmate similarity scores in -/+ category compared to the +/+ category ($t_{30} = 1.98$, $p = 0.057$, $d = 0.36$, CI = [0.004 ± 0.005], two-tailed paired samples *t*-test). Notes: ** $p < .01$, * $p < .05$, ~ $p < .10$. No correction for multiple comparisons was applied given the a priori predictions for CA3/DG. Data are presented as mean values ± S.E.M. and all data reflect $n = 31$ independent participants. Source data are provided as a Source Data file.

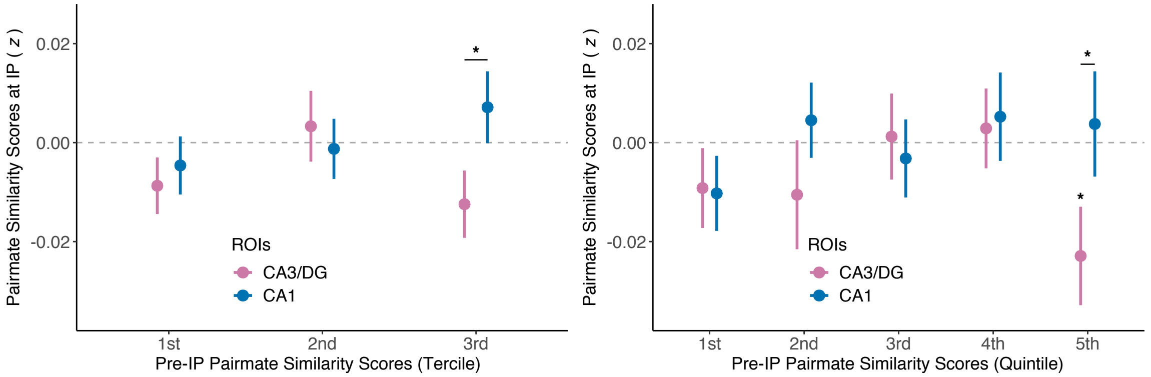

Supplementary Figure 3. Pairmate similarity scores at the inflection point (IP) as a function of relative pairmate similarity scores at the pre-inflection point (pre-IP) (related to Fig. 3c). Left: pre-IP pairmate similarity scores binned into terciles (from lowest to highest). Right: pre-IP pairmate similarity scores binned into quintiles (from lowest to highest). For both analyses, binning was performed within-subject and separately for CA3/dentate gyrus (CA3/DG) and CA1. When binned by terciles or quintiles, pairmate similarity scores in CA3/dentate gyrus were significantly lower than in CA1 at the highest pre-IP bin (terciles: t_{30} = -2.22, *p* = .034, *d* = 0.40, CI = [-0.020 ± 0.018]; quintiles: t_{30} = -2.18, *p* = .037, *d* = 0.39, CI = [-0.027 ± 0.025]; two-tailed paired samples *t*-tests). When binned by terciles, pairmate similarity scores in CA3/dentate gyrus were marginally below 0 for the highest pre-IP bin (*t*-test vs. 0: t_{30} = -1.83, *p* = .077, *d* = 0.33, CI = [-0.012 ± 0.014]; two-tailed one sample t-test). When binned by quintiles, pairmate similarity scores in CA3/dentate gyrus were significantly below 0 for the highest pre-IP bin (two-tailed one sample *t*-test vs. 0: t_{30} = -2.30, *p* = 028, *d* = 0.41, CI = [-0.023 ± 0.020]). Note: * $p < .05$. No correction for multiple comparisons was applied given the a priori predictions for CA3/DG. Data are presented as mean values ± S.E.M. and all data reflect *n* = 31 independent participants. Source data are provided as a Source Data file.

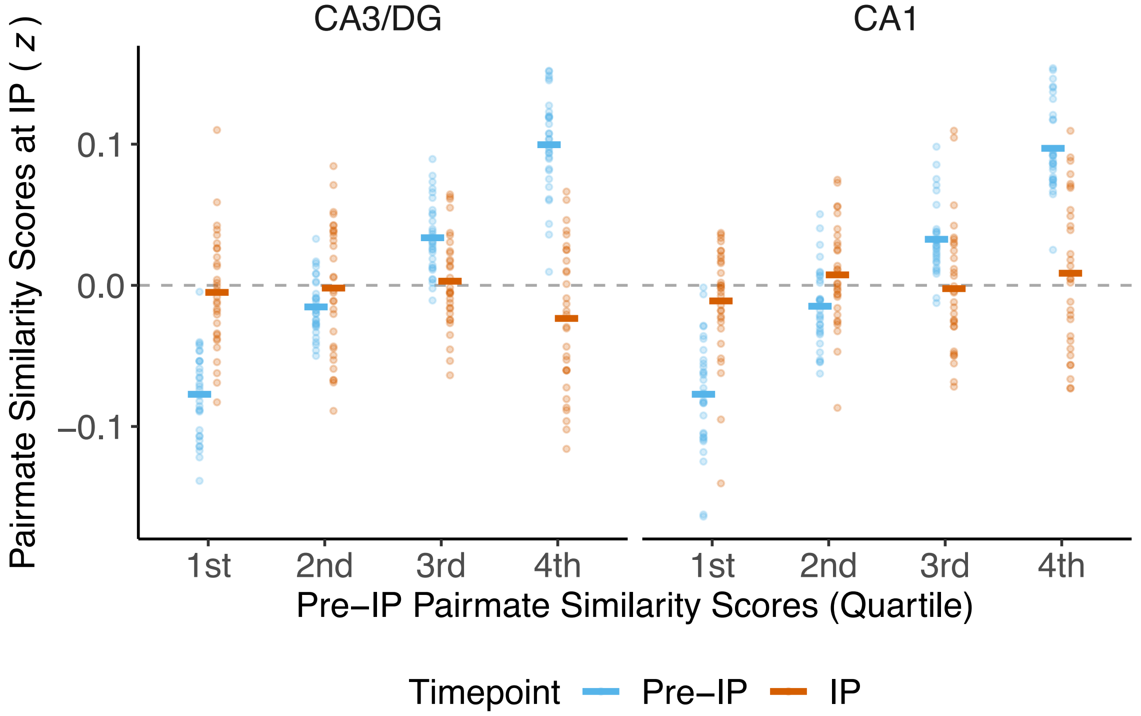

Supplementary Figure 4. Distributions of pairmate similarity scores at the pre-inflection point (pre-IP) and inflection point (IP), as a function of pre-IP similarity (related to Fig. 3c). Mean pairmate similarity scores at the pre-IP were binned into quartiles, separately for each subject and for CA3/dentate gyrus (CA3/DG) and CA1. Pre-IP data (blue dots) show the distribution (across subjects) of the pairmate similarity scores at each pre-IP bin. IP data (orange dots) show the distribution (across subjects) of pairmate similarity scores at the inflection point as a function of the pre-IP pairmate similarity level (1st quartile = lowest pre-IP similarity, 4th quartile = highest pre-IP similarity). Note: direct comparison of pre-IP versus IP values at each bin is not statistically valid given that the pre-IP data, but not the IP data, were binned by value (quartiles). Notes: all data reflect n = 31 independent participants. Source data are provided as a Source Data file.

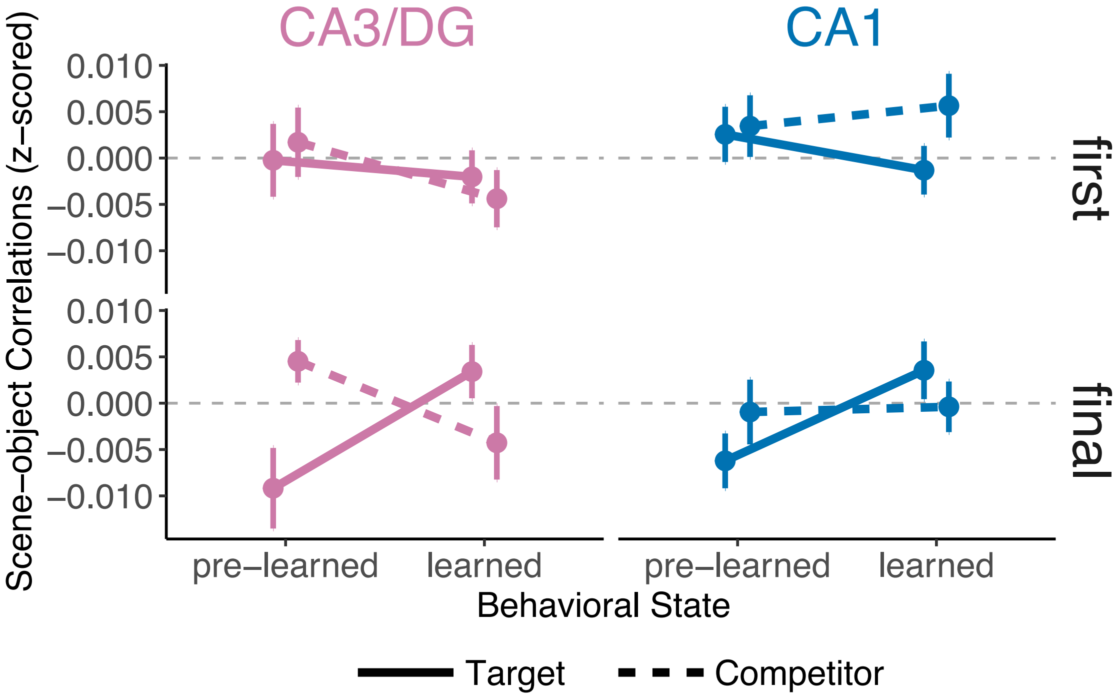

Supplementary Figure 5. Scene-object similarity as a function of object relevance (target, competitor), ROI (CA3/dentate gyrus, CA1), behavioral state (pre-learned, learned), and object run (first, final). The 3-way interaction between object relevance, behavioral state, and object run was not significant for CA3/dentate gyrus (CA3/DG: $F_{1,30} = 3.14$, $p = 0.086$, $\eta^2 = 0.01$, repeated measures ANOVA) or for CA1 ($F_{1,30} = 3.90$, $p = 0.057$, $\eta^2 = 0.01$, repeated measures ANOVA). When considering each object run separately, CA3/dentate gyrus exhibited a significant interaction between object relevance and behavioral state for data from the final object run ($F_{1,30} = 12.65$, $p = 0.001$, $\eta^2 = 0.07$, repeated measures ANOVA), but not from the first object run ($F_{1,30} = 0.48$, $p = 0.495$, $\eta^2 = 0.003$, repeated measures ANOVA). CA1 did not exhibit a significant interaction between object relevance and behavioral state for data from either object run (first run: $F_{1,30} = 1.34$, $p = 0.255$, $\eta^2 = 0.01$; final run: $F_{1,30} = 3.85$, $p = 0.059$, $\eta^2 = 0.18$; repeated measures ANOVAs). Note: no correction for multiple comparisons was applied given the a priori predictions for CA3/DG. Data are presented as mean values ± S.E.M. and all data reflect $n = 31$ independent participants. Source data are provided as a Source Data file.

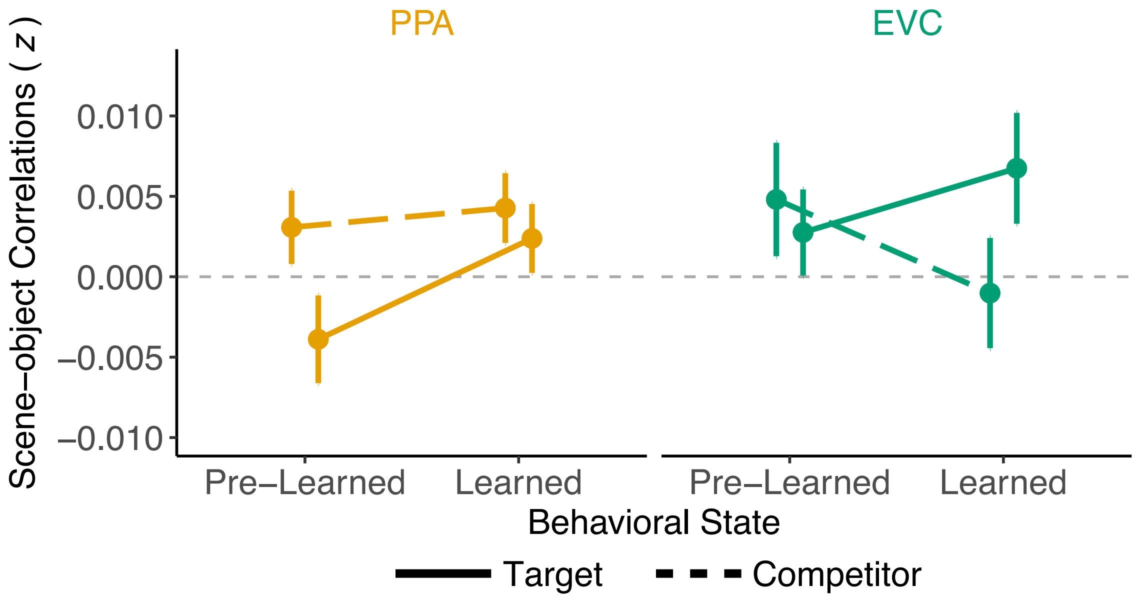

Supplementary Figure 6. Scene-object similarity in PPA and EVC as a function of behavioral state. Scene-object similarity as a function of object relevance (target, competitor), ROI (PPA, yellow; EVC, green), and behavioral state (pre-learned round, learned round). There was no significant interaction between behavioral state and object relevance for either ROI (PPA: $F_{1,30} = 1.97$, $p = 0.170$, $\eta^2 = 0.01$; EVC: $F_{1,30} = 3.23$, $p = 0.082$, $\eta^2 = 0.02$, repeated measures ANOVAs). Note: No correction for multiple comparisons was applied. Data are presented as mean ± S.E.M. and all data reflect $n = 31$ independent participants. Source data are provided as a Source Data file.

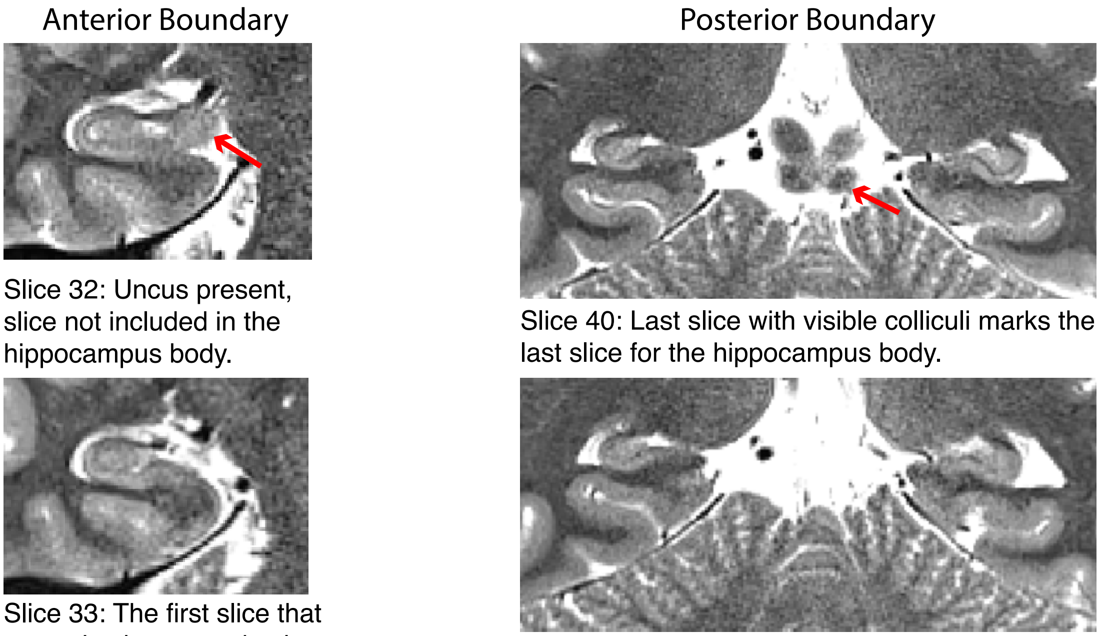

Slice 32: Uncus present, slice not included in the hippocampus body.

Slice 33: The first slice that uncus is absent marks the the first slice of the hippocampus body.

Slice 40: Last slice with visible colliculi marks the last slice for the hippocampus body.

Slice 41: Colliculi disappeared, slice not included in the hippocampus body.

Supplementary Figure 7. Illustration of anterior and posterior boundaries for the hippocampal body from a sample participant.
