# Supplementary material for: Abrupt hippocampal remapping signals resolution of memory interference
Source: Nat Commun. 2021 Aug 10;12:4816. doi: 10.1038/s41467-021-25126-0 (PMC8355182; doi:10.1038/s41467-021-25126-0)
Supplement: Supplementary file 3 — Reporting Summary [file 41467_2021_25126_MOESM3_ESM.pdf]

nature portfolio

Corresponding author(s): Guo Wanjia: wanjiag@uoregon.edu
Brice A. Kuhl, Ph.D.: bkuhl@uoregon.edu

Last updated by author(s): Jun 21, 2021

Reporting Summary

Nature Portfolio wishes to improve the reproducibility of the work that we publish. This form provides structure for consistency and transparency in reporting. For further information on Nature Portfolio policies, see our Editorial Policies and the Editorial Policy Checklist.

Statistics

For all statistical analyses, confirm that the following items are present in the figure legend, table legend, main text, or Methods section.

n/a	Confirmed	
☐	☒	The exact sample size (*n*) for each experimental group/condition, given as a discrete number and unit of measurement
☐	☒	A statement on whether measurements were taken from distinct samples or whether the same sample was measured repeatedly
☐	☒	The statistical test(s) used AND whether they are one- or two-sided<br>*Only common tests should be described solely by name; describe more complex techniques in the Methods section.*
☒	☐	A description of all covariates tested
☐	☒	A description of any assumptions or corrections, such as tests of normality and adjustment for multiple comparisons
☐	☒	A full description of the statistical parameters including central tendency (e.g. means) or other basic estimates (e.g. regression coefficient) AND variation (e.g. standard deviation) or associated estimates of uncertainty (e.g. confidence intervals)
☐	☒	For null hypothesis testing, the test statistic (e.g. *F*, *t*, *r*) with confidence intervals, effect sizes, degrees of freedom and *P* value noted<br>*Give P values as exact values whenever suitable.*
☒	☐	For Bayesian analysis, information on the choice of priors and Markov chain Monte Carlo settings
☒	☐	For hierarchical and complex designs, identification of the appropriate level for tests and full reporting of outcomes
☐	☒	Estimates of effect sizes (e.g. Cohen's *d*, Pearson's *r*), indicating how they were calculated

Our web collection on statistics for biologists contains articles on many of the points above.

Software and code

Policy information about availability of computer code

Data collection	The experiment task was presented using PsychoPy (v.3.0) within Python. Brain imaging was acquired on a Siemens 3T Skyra MRI system in the Lewis Center for Neuroimaging at the University of Oregon.
Data analysis	MRI data was firstly preprocessed with fmriprep (v1.5.0) and ANTs (v2.2.0) and FSL (v5.0.9) in Nipype (v1.2.2) environment with Python (v3.7.0). Data analysis was performed in R (v3.5.0) and its associated libraries.

For manuscripts utilizing custom algorithms or software that are central to the research but not yet described in published literature, software must be made available to editors and reviewers. We strongly encourage code deposition in a community repository (e.g. GitHub). See the Nature Portfolio guidelines for submitting code & software for further information.

Data

Policy information about availability of data

All manuscripts must include a data availability statement. This statement should provide the following information, where applicable:

- Accession codes, unique identifiers, or web links for publicly available datasets
- A description of any restrictions on data availability
- For clinical datasets or third party data, please ensure that the statement adheres to our policy

The MRI data generated in this study have been deposited on Openneuro.org (DOI: 10.18112/openneuro.ds003707.v1.0.0). The behavioral data generated in this study have been deposited on osf.io (DOI: 10.17605/OSF.IO/VPQ2X).

Field-specific reporting

Please select the one below that is the best fit for your research. If you are not sure, read the appropriate sections before making your selection.

☒ Life sciences ☐ Behavioural & social sciences ☐ Ecological, evolutionary & environmental sciences

For a reference copy of the document with all sections, see nature.com/documents/nr-reporting-summary-flat.pdf

Life sciences study design

All studies must disclose on these points even when the disclosure is negative.

Sample size	36 participants were enrolled in the study. After exclusions due to poor performance or excessive motion in the scanner (see below), 31 participants remained in the MRI analysis. Although a relevant effect size was not available in advance (due to the novel analyses), the sample size was designed to be approximately 2 times more than what was used in a previous fMRI study with similar design (Favila et al. 2016), and consistent with other similar fMRI studies.
Data exclusions	5 participants were excluded from the analysis based on meeting either or both of the following criteria: (1) excessive motion (>3.5mm) in the scanner (n=1); (2) behavioral performance lead to less than 8 pairs of usable stimuli (n=4). Exclusion criteria were per-established based on other fMRI studies and pilot data.
Replication	A single fMRI experiment was conducted and is reported. A behavioral pilot version of the experiment was conducted in order to confirm that human subjects were able to successfully learn the associations in the experiment. The behavioral data from the fMRI experiment replicated the pattern of data from the behavioral pilot experiment.
Randomization	For each participants, the association between scene and object were semi-random with the limitation that similar scenes were assigned to similar objects. The display of each association was randomized for each block for each participant. The location of the choices in the associative tests were also randomized for each trial. The appearance of novel lure objects was also randomized for each block for each subject.
Blinding	Data collection and analysis were not performed blind to the conditions of the experiments. The lead author of the study was responsible for both data collection and analysis and blinding was therefore impractical. Additionally, because all experimental manipulations were performed within subjects (varying on a trial-by-trial basis), blind data collection was not feasible or necessary.

Reporting for specific materials, systems and methods

We require information from authors about some types of materials, experimental systems and methods used in many studies. Here, indicate whether each material, system or method listed is relevant to your study. If you are not sure if a list item applies to your research, read the appropriate section before selecting a response.

Materials & experimental systems

n/a	Involved in the study	
☒	☐	Antibodies
☒	☐	Eukaryotic cell lines
☒	☐	Palaeontology and archaeology
☒	☐	Animals and other organisms
☐	☒	Human research participants
☒	☐	Clinical data
☒	☐	Dual use research of concern

Methods

n/a	Involved in the study	
☒	☐	ChIP-seq
☒	☐	Flow cytometry
☐	☒	MRI-based neuroimaging

Human research participants

Policy information about studies involving human research participants

Population characteristics	Thirty-six participants (21 female; mean age = 23.69 yrs, range = 18 – 34 yrs) were enrolled in the experiment.
Recruitment	Recruited via advertisement (fliers and emails). Because all experimental manipulations were within-participant, there was no opportunity for self-selection biases that contributed to differences between conditions.
Ethics oversight	Institutional Review Board at the University of Oregon

Note that full information on the approval of the study protocol must also be provided in the manuscript.

Magnetic resonance imaging

Experimental design

Design type	task-based, event-related design
Design specifications	For each participant, 8 blocks of functional MRI data were collected. Each block includes 78 trials of scene/object display (length: 4s for each trial) and 4 trials of odd/even number display (length: 2.5s for each trial).
Behavioral performance measures	Accuracy and response times were recorded. Mean and standard error of accuracy, as well as d-prime were calculated to ensure that the subjects were performing the task as expected.

Acquisition

Imaging type(s)	Functional MRI and Structural MRI.
Field strength	3T
Sequence & imaging parameters	Functional data were acquired with a T2*-weighted echo-planar imaging sequence (repetition time = 2000 ms, echo time = 36 ms, flip angle = 90°, 72 slices, 1.7x1.7x1.7mm voxels).<br>Anatomical scans includes a whole-brain high-resolution T1-weighted magnetization prepared rapid acquisition gradient echo anatomical volume (1x1x1mm voxels) and a high-resolution (coronal direction) T2-weighted scan (0.43x0.43x2mm voxels, aligned with hippocampus).
Area of acquisition	Whole-brain was used for T1; T2 was collected with partial-brain coverage that prioritized hippocampus. Functional scans was collected with partial-brain coverage that prioritized full coverage of the hippocampus and early visual cortex.

Diffusion MRI ☐ Used ☒ Not used

Preprocessing

Preprocessing software	MRI data was preprocessed with fmriprep (v1.5.0) and ANTs (v2.2.0) and FSL (v5.0.9) in Nipype (v1.2.2) environment with Python (v3.7.0).
Normalization	No normalization was performed. Participants were analyzed in their own native T1 space.
Normalization template	fMRI data were not normalized to a standard space because all analyses were performed in native space. fMRI data were only aligned at the level of regions of interest, not at the level of individual voxels.
Noise and artifact removal	A deformation field to correct for susceptibility distortions was estimated based on two echo-planar imaging (EPI) references with opposing phase-encoding directions, using 3dQwarp, AFNI. Based on the estimated susceptibility distortion, an unwarped BOLD reference was calculated for a more accurate co-registration with the anatomical reference. Framewise displacement, xyz translation and xyz rotation were represented as nuisance regressors in GLM analysis.
Volume censoring	Volume censoring was not conducted.

Statistical modeling & inference

Model type and settings	Representational similarity analyses were applied to first-level GLMs that were calculated using a Double-Gamma HRF with temporal derivatives. No second-level model was calculated or used in the study.
Effect(s) tested	To compare pairmate similarity scores and other measures across ROIs and learning states, repeated measures ANOVAs and paired-samples t-tests were used. To test whether pairmate similarity scores and other measures were significantly positive or negative (i.e., above/below 0), one-sample t-tests were used.

Specify type of analysis: ☐ Whole brain ☒ ROI-based ☐ Both

Anatomical location(s)	Probabilistic atlases were used for EVC and PPA ROI. Automatic Segmentation of Hippocampal Subfields (ASHS) toolbox was used for hippocampus subfields ROIs.
Statistic type for inference (See Eklund et al. 2016)	Analyses were performed in a small set of a priori, anatomical ROIs. Thus, the inference was not voxelwise or clusterwise.
Correction	Corrections for multiple comparisons were not applied. Although we included four regions of interest in many of the analyses, we had a specific a priori interest in a single region of interest (CA3/dentate gyrus) and all of our main conclusions are based on this region of a priori interest which was consistently implicated across independent analyses. The inclusion of other ROIs was for the sake of comparison.

Models & analysis

n/a	Involved in the study
☒	☐ Functional and/or effective connectivity
☒	☐ Graph analysis
☒	☐ Multivariate modeling or predictive analysis
